# Compact Belief Rule Base Learning for Classification with Evidential Clustering †

**DOI:** 10.3390/e21050443

**Published:** 2019-04-28

**Authors:** Lianmeng Jiao, Xiaojiao Geng, Quan Pan

**Affiliations:** School of Automation, Northwestern Polytechnical University, Xi’an 710072, China

**Keywords:** rule-based classification, belief function theory, evidential C-means, evidential partition entropy

## Abstract

The belief rule-based classification system (BRBCS) is a promising technique for addressing different types of uncertainty in complex classification problems, by introducing the belief function theory into the classical fuzzy rule-based classification system. However, in the BRBCS, high numbers of instances and features generally induce a belief rule base (BRB) with large size, which degrades the interpretability of the classification model for big data sets. In this paper, a BRB learning method based on the evidential C-means clustering (ECM) algorithm is proposed to efficiently design a compact belief rule-based classification system (CBRBCS). First, a supervised version of the ECM algorithm is designed by means of weighted product-space clustering to partition the training set with the goals of obtaining both good inter-cluster separability and inner-cluster pureness. Then, a systematic method is developed to construct belief rules based on the obtained credal partitions. Finally, an evidential partition entropy-based optimization procedure is designed to get a compact BRB with a better trade-off between accuracy and interpretability. The key benefit of the proposed CBRBCS is that it can provide a more interpretable classification model on the premise of comparative accuracy. Experiments based on synthetic and real data sets have been conducted to evaluate the classification accuracy and interpretability of the proposal.

## 1. Introduction

Pattern classification is a popular research field in artificial intelligence. The main purpose of classification is to assign the objects, represented by feature vectors to predefined group of classes [1]. In the past five decades, a variety of classification techniques, such as K-nearest neighbors (K-NN) [2], decision trees (DT) [3], support vector machines (SVM) [4], rule-based classification (RBC) [5], have been proposed. Among these methods, the RBC not only obtains the advantage in classification result interpreting, but also can be easily enhanced by adding new rules from experts’ domain knowledge. As one of the most representative RBC methods, a fuzzy rule-based classification system (FRBCS) [5,6] has been developed by incorporating fuzzy sets [7]. The FRBCS is widely used because it can build a linguistic model interpretable to users. It has been successfully applied to many classification tasks where model interpretability is important, such as terrain classification [8], intrusion detection [9], fault prediction [10], disease diagnosis [11], and target recognition [12].

However, in real-world complex systems, different types of uncertainty (such as fuzziness, imprecision and incompleteness) may coexist. The FRBCS, which is based on fuzzy set theory, cannot model those imprecise or incomplete information effectively. The belief function theory, proposed by Dempster [13] and Shafer [14] et al., provides a powerful framework for uncertain modeling and reasoning. As fuzzy set theory and belief function theory are suited to dealing with different types of uncertainty, some researchers have investigated the relationship between them and suggested different integrating ways [15,16,17,18]. Among them, in [18], a belief rule-based classification system (BRBCS) was developed by extending the FRBCS within the framework of belief function theory to address imprecise or incomplete information in complex classification problems. In contrast to the traditional fuzzy rule, the new belief rule assigns the consequent part with a belief distribution structure, so that different kinds of uncertain information existing in the training set can be well characterized. Besides, to reduce the risk of misclassification in noisy conditions, the classification of a query pattern is made by combining all the activated belief rules. In many situations, this method is found experimentally to yield better classification accuracy and robustness than the FRBCS using the same information.

Rule learning is the most important issue in developing the BRBCS. In [18], a heuristic belief rule base (BRB) learning method was developed by defining belief rules based on fuzzy-grid partitions of the feature space and individuals of the training patterns, and the resulting BRB can provide an accurate mapping between the feature space and the class space. However, with this method, higher numbers of instances and features generally induce a BRB with larger size. This may lead to a large rule base for big data set, which degrades the interpretability of the classification model. Motivated by the above consideration, in this paper, a compact belief rule-based classification system (CBRBCS) is developed for a better trade-off between accuracy and interpretability (A preliminary version of some of the ideas introduced here was presented in [19,20]. The present paper is a deeply revised and extended version of this work, with several new results.). We propose to learn a compact BRB based on partitions of the training set realized with clustering techniques. The evidential C-mean (ECM) algorithm [21], which extended the fuzzy C-mean (FCM) algorithm [22] within the framework of belief functions, is used for its capability to address imprecise and partial information existing in the observed data. As belief rules are constructed based on credal partitions of the training set, this method can reduce the number of generated rules greatly. The main contributions of this paper are as follows:

1. A supervised version of the ECM algorithm is designed by means of weighted product-space clustering to take into account the class labels, which can obtain credal partitions with both good inter-cluster separability and inner-cluster pureness.
2. A systematic method is developed to construct belief rules (composed of the antecedent part, the consequent class, and the rule weight) based on credal partitions of the training set.
3. A two-objective optimization procedure based on both the mean squared error and the evidential partition entropy is designed to get a compact BRB with a better trade-off between accuracy and interpretability.

Two types of experiments using both synthetic and real data sets have been developed to evaluate the performance of the proposed CBRBCS. In the synthetic data test, a two-dimensional four-class synthetic data set was designed to illustrate the interest of the compact BRB learning under different parameter settings. In the real data test, 20 data sets varying greatly in the number of instances, features, and classes were selected from the UCI Machine Learning Repository [23] for evaluation. The comparison methods cover the traditional BRBCS, as well as some of the most representative classifiers, including K-NN, C4.5, SVM, and FRBCS. The reported results show that the proposed CBRBCS can obtain competitive performance compared with those representative classifiers for a variety of real tasks involving different data conditions, and get a better trade-off between accuracy and interpretability than the traditional BRBCS. Therefore, it provides a better choice of classification technique for those problems where both high accuracy and interpretability are needed.

The rest of the paper is organized as follows. In Section 2, some preliminaries of the related theories and methods are reviewed. The compact BRB learning with ECM is developed in Section 3. The experiments to evaluate the performance of the proposed method are reported in Section 4. At last, Section 5 concludes the paper.

## 2. Background

In this section, we provide some preliminaries of the related theories and methods. We first introduce some basic concepts of the belief function theory in Section 2.1. After that, we give overviews of the BRBCS classification method and the ECM clustering method in Section 2.2 and Section 2.3, respectively. The used symbols and their definitions are listed in Table 1 to facilitate reading.

### 2.1. Basics of the Belief Function Theory

In belief function theory [13,14], a problem domain is represented by a finite set $\Omega =\{\omega_{1},\omega_{2},\cdots ,\omega_{C}\}$ called the *frame of discernment*. A *mass function* expressing the belief committed to the elements of $2^{\Omega }$ by a given source of evidence is a mapping function *m*: $2^{\Omega }\to \left[0,1\right]$, such that

(1) $$\sum_{A\in 2^{\Omega }}m(A)=1.$$

Elements A⊆Ω with m(A)>0 are called the *focal sets* of the mass function *m*. The mass function *m* has several special cases, which represent different types of information. A mass function is said to be

- *normal*, if m(∅)=0. Otherwise, it is *subnormal*, and m(∅) is interpreted as a mass of belief given to the hypothesis that ω might not lie in Ω.
- *Bayesian*, if all its focal sets are singletons. In this case, the mass function reduces to the precise probability distribution;
- *certain*, if the whole mass is allocated to a unique singleton. This corresponds to a situation of complete knowledge;
- *vacuous*, if the whole mass is allocated to Ω. This situation corresponds to complete ignorance.

After representing the available pieces of evidence as mass functions, one often needs to combine several mass functions into a single one. *Dempster’s rule* is the most popular way to combine several distinct pieces of evidence. The Dempster’s rule of combination of two normal mass functions $m_{1}$ and $m_{2}$ defined on the same frame of discernment Ω is given by

(2) $$m_{1}⊕m_{2}\left(A\right)=\left\{\begin{array}{ll} 0, & A=\emptyset \\ \frac{\sum_{B\cap C=A}m_{1}\left(B\right)m_{2}\left(C\right)}{1-\sum_{B\cap C=\emptyset }m_{1}\left(B\right)m_{2}\left(C\right)}, & A\in 2^{\Omega }\backslash \emptyset . \end{array}\right.$$

Dempster’s rule of combination is both commutative and associative.

### 2.2. Belief Rule-Based Classification System (BRBCS)

The BRBCS is composed of two components, the BRB and the belief reasoning method (BRM) [18]. The BRB is first constructed to establishes a mapping between the feature space and the class space, and then the BRM is used to classify a query pattern based on the constructed BRB.

For an *M*-class (denoted as $C=\{c_{1},c_{2},\cdots ,c_{M}\}$) classification problem with *P* features, the BRB consists of a collection of belief rules defined as follows:

$$\begin{array}{ll} R^{j}: & \mathrm{If}\;x_{1}\;\mathrm{is}\;A_{1}^{j}\;\mathrm{and}\;x_{2}\;\mathrm{is}\;A_{2}^{j}\;\mathrm{and}\cdots \;\mathrm{and}\;x_{P}\;\mathrm{is}\;A_{P}^{j},\mathrm{then}\;\mathrm{class}\;\mathrm{is}\;C^{j}=\left\{\left(c_{1},\beta_{1}^{j}\right),\cdots ,\left(c_{M},\beta_{M}^{j}\right)\right\},\\ & \mathrm{with}\;\mathrm{rule}\;\mathrm{weight}\;\theta^{j},\;\;j=1,2,\cdots , \end{array}$$

where $x_{1},x_{2},\cdots ,x_{P}$ represent the antecedent features and $A^{j}=\left(A_{1}^{j},A_{2}^{j},\cdots ,A_{P}^{j}\right)$ is the antecedent part of the belief rule $R^{j}$ with each $A_{p}^{j}$ belonging to fuzzy partitions $\left\{A_{p,1},A_{p,2},\cdots ,A_{p,n_{p}}\right\}$ associated with *p*-th feature, p=1,⋯,P. $\beta_{k}^{j}$ is the belief degree that input data $x=(x_{1},x_{2},\cdots ,x_{P})$ belongs to $c_{k}$, k=1,⋯,M. In the belief structure, the consequence may be incomplete, i.e., $\sum_{k=1}^{M}\beta_{k}^{j}\leq 1$, and the left belief $1-\sum_{k=1}^{M}\beta_{k}^{j}$ denotes the degree of global ignorance about the consequence. The rule weight $\theta^{j}$ with $0\leq \theta^{j}\leq 1$, characterizes the certainty grade of the belief rule $R^{j}$.

The BRB can be learned from training data or derived from expert knowledge [24]. In [18], a heuristic BRB learning method was developed based on fuzzy-grid partitions of the feature space. To generate the BRB, this method uses the following steps:

**Step 1:**  *Partition of the feature space*.
  A fuzzy-grid-based method is used to divide the *P*-dimensional feature space into $\prod_{p=1}^{P}n_{p}$ fuzzy regions, with $n_{p}$ being the number of partitions for *p*-th feature.
**Step 2:**  *Generation of the consequent class for each fuzzy region*.
  Each training pattern is assigned to the fuzzy region with the greatest matching degree, and those patterns assigned to the same fuzzy region are fused to get the consequent class.
**Step 3:**  *Generation of the rule weights*.
  The rule weights are determined by two measures called confidence and support jointly.

Once the BRB is generated, the BRM is used to classify a query pattern by combining the consequent parts of all the activated belief rules (refer to [18] for details of this reasoning method).

### 2.3. Evidential C-Means (ECM)

In [21], an ECM algorithm was proposed to derive credal partitions from object data. In this algorithm the class membership of an object $x_{i}$ is represented by a mass function $m_{i}$ defined on the power set of a given frame of discernment $\Omega =\{\omega_{1},\omega_{2},\cdots ,\omega_{C}\}$. The credal partitions of *N* observed data $\left\{x_{1},x_{2},\cdots ,x_{N}\right\}\in R^{P}$ are then defined as the *N*-tuple $M=(m_{1},m_{2},\cdots ,m_{N})$. It can be seen as a general model of partitioning, where:

- when each $m_{i}$ is a *certain* mass function, then *M* defines the conventional, crisp partitions of the set of objects;
- when each $m_{i}$ is a *Bayesian* mass function, then *M* specifies the fuzzy partitions, as defined by Bezdek [22].

For each object $x_{i}$, the quantities $m_{ij}=m_{i}\left(A_{j}\right)$$\left(A_{j}\subseteq \Omega ,A_{j}\neq \emptyset \right)$ are determined in such a way that the mass of belief $m_{ij}$ is low (high) when the distance $d_{ij}$ between object $x_{i}$ and set $A_{j}$ is high (low). The distance between object $x_{i}$ and set $A_{j}$ is calculated by $d_{ij}=∥x_{i}-{\bar{v}}_{j}∥$, where ${\bar{v}}_{j}$ is the barycenter of the centers associated with the classes composing $A_{j}$. Denoting $v_{k}$ the center of the single cluster $\omega_{k}$, the barycenter ${\bar{v}}_{j}$ is calculated as

(3) $${\bar{v}}_{j}=\frac{1}{|A_{j}|}\sum_{k=1}^{C}s_{kj}v_{k}\;\;\mathrm{with}\;\;s_{kj}=\left\{\begin{array}{c} 1,\;\mathrm{if}\;\omega_{k}\in A_{j}\\ 0,\;\mathrm{otherwise} \end{array}.\right.$$

Finally, the objective function used to derive the credal partition matrix *M* of size $2^{C}\times N$ and the cluster center matrix *V* of size C×P, is given by

(4) $$J_{\mathrm{ECM}}\left(M,V\right)=\sum_{i=1}^{N}\sum_{\left\{j/A_{j}\subseteq \Omega ,A_{j}\neq \emptyset \right\}}|A_{j}{|}^{\alpha }m_{ij}^{\beta }d_{ij}^{2}+\sum_{i=1}^{N}\delta^{2}m_{i\emptyset }^{\beta },$$

subject to

(5) $$\sum_{\left\{j/A_{j}\subseteq \Omega ,A_{j}\neq \emptyset \right\}}m_{ij}+m_{i\emptyset }=1,\;\;\forall i=1,\cdots ,N,$$

where β>1 is a weighting exponent to control the fuzziness of the partition, α≥0 is a weighting exponent to control the degree of penalization for the subsets in Ω of high cardinality, δ>0 is a distance to control the amount of data considered to be outliers, and $m_{i\emptyset }$ denotes $m_{i}\left(\emptyset \right)$, the mass that the class of object $x_{i}$ does not lie in Ω. This objective function is minimized using an iterative algorithm, which alternatively optimizes the credal partition matrix *M* and the cluster center matrix *V*.

## 3. Compact BRB Learning with ECM

**Figure 1:**
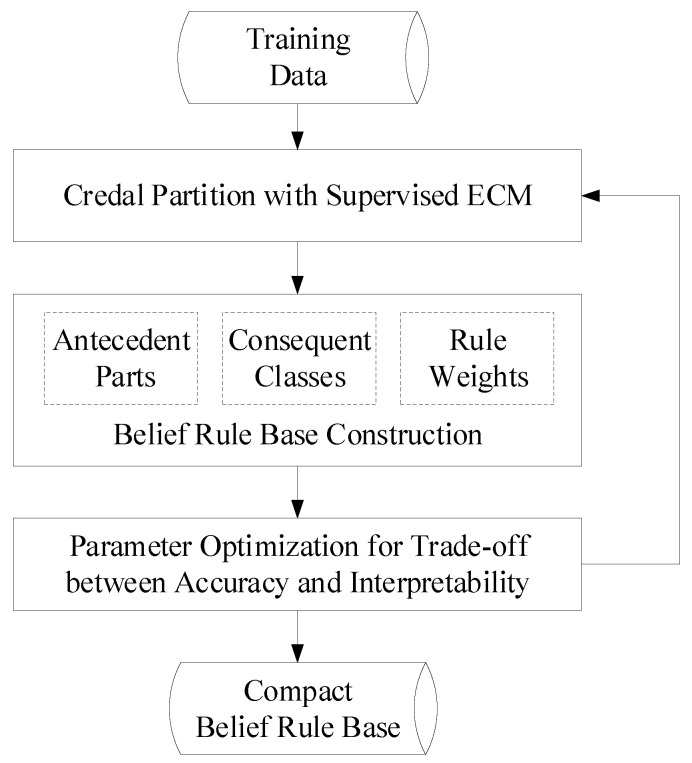
Flow diagram of the compact BRB learning with ECM.

As reviewed in Section 2.2, in the traditional BRB learning method, belief rules are defined based on fuzzy-grid partitions of the feature space and individuals of the training instances. This may lead to a large rule base for big data set with large numbers of instances and features, which degrades the interpretability of the classification model. In this section, we propose to learn a compact BRB based on partitions of the training set realized with clustering techniques. The ECM algorithm is used here to incorporate the additional degrees of freedom and information obtained from the derived credal partitions, in the BRBCS. The flow diagram of the proposed compact BRB learning with ECM is shown in Figure 1. First, the ECM algorithm operates in a supervised way in Section 3.1 by means of weighted product-space clustering with the goals of obtaining credal partitions with both good inter-cluster separability and inner-cluster pureness. Then, Section 3.2 shows how to construct belief rules based on credal partitions of the training set. Finally, a two-objective optimization procedure is designed in Section 3.3 to get a compact BRB with a better trade-off between accuracy and interpretability.

### 3.1. Credal Partition with Supervised ECM

In typical classification problems, a set of *N* labeled patterns $T=\{\left(x_{1},c^{\left(1\right)}\right)$, $\left(x_{2},c^{\left(2\right)})\right\}$, $\cdots ,(x_{N},c^{\left(N\right)})\}$ with input vectors $x_{i}\in R^{P}$ and class labels $c^{\left(i\right)}\in \left\{c_{1},c_{2},\cdots ,c_{M}\right\}$ are available, and the problem is to classify a query pattern y based on the training set T. In contrast to unsupervised clustering problems which only consider the *inter-cluster separability*, a good partition of labeled patterns should also take into account the *inner-cluster pureness*. For this purpose, we cluster the *N* labeled patterns in the following weighted product space

(6) z=(x×Wc),

where W≥0 controls the weight of class labels in clustering process. If W=0, it just reduces to the unsupervised clustering, and as W→∞, the resulting clusters are the same with those obtained by dividing the training set only based on the class labels directly. A suggested choice of *W* for balancing the effects of feature values and class values is

(7) $$W=\sqrt{\frac{\sum_{p=1}^{P}\sigma_{p}^{2}}{\sigma_{c}^{2}}},$$

where $\sigma_{p}^{2}$ is the variance of *p*-th feature values, p=1,2,⋯,P, and $\sigma_{c}^{2}$ is the variance of class values.

With given weight *W* and number of clusters *C*, the ECM clustering algorithm operates in the above supervised way to discover credal partitions of the training set T in the weighted product space. Two practical issues concerning the credal partitions are further considered to be follows.

- *Limiting the number of credal partitions*. By minimizing the objective function displayed as Equation (4), a maximum number of $2^{C}$ credal partitions can be obtained. However, those credal partitions composed of many classes are quite difficult to interpret and are usually also less important in practice. Therefore, in order to learn a compact BRB, we constrain the focal sets to be either Ω, or to be composed of at most two classes, thereby reducing the maximum number of credal partitions from $2^{C}$ to $\left(C^{2}+C\right)/2+2≜F\left(C\right)$.
- *Discarding the outlier cluster*. In ECM, the training patterns assigned to empty set are considered to be outliers, which are adverse to classification. Thus, we only construct belief rules based on the left F(C)−1 credal partitions associated with non-empty focal sets.

### 3.2. Belief Rule Base Construction

As shown in Section 2.2, each belief rule is composed of three components, namely the antecedent part, the consequent class, and the rule weight. In the following part, we will show how to construct belief rules from these three aspects based on credal partitions of the training set obtained previously.

#### 3.2.1. Antecedent Parts Generation

From the obtained credal partition matrix *M*, whose ij-th element $m_{ij}\to \left[0,1\right]$ is the membership degree of the data $x_{i}$ in partition *j*, it is possible to extract the fuzzy sets in the antecedent parts of the belief rules. One-dimensional antecedent fuzzy sets $A_{p}^{j}$ are obtained from the multidimensional credal partition *M* by point-wise projection [25] onto the space of the antecedent features $x_{p}$, p=1,2,⋯,P:

(8) $$\mu_{A_{p}^{j}}\left(x_{ip}\right)={\mathrm{proj}}_{p}\left(m_{ij}\right).$$

With the above point-wise defined membership, a continuous membership function $\mu_{A_{p}^{j}}\left(x\right)$ for fuzzy sets $A_{p}^{j}$ can be approximated. Several types of functions such as triangular, trapezoidal, or Gaussian, can be used. In this work we choose the Gaussian membership function of the form

(9) $$\mu_{A_{p}^{j}}\left(x\right)=f\left(x;{\bar{v}}_{jp},\sigma_{jp}\right)=e^{\left(-\frac{{\left(x-{\bar{v}}_{jp}\right)}^{2}}{2\sigma_{jp}^{2}}\right)},$$

where ${\bar{v}}_{jp}$ is the mean value calculated as Equation (3), and $\sigma_{jp}$ is the standard variance to be estimated.

In this way, for each credal partition *j*, j=1,2,⋯,F(C)−1, a series of fuzzy sets $A_{1}^{j},A_{2}^{j},\cdots ,A_{P}^{j}$ can be defined on the antecedent features with Gaussian membership functions, which finally constitute the antecedent part of belief rule $R^{j}$.

#### 3.2.2. Consequent Classes Generation

Based on the credal partition matrix *M*, the training set T can be divided into F(C)−1 groups by assigning each pattern to the partition with highest mass:

(10) $$T^{j}=\left\{\left(x_{i},c^{\left(i\right)}\right)|m_{ij}=\max_{k}m_{ik},i=1,\cdots ,N\right\},\;\;j=1,2,\cdots ,F\left(C\right)-1.$$

The training subsets $T^{j}$ for j=1,2,⋯,F(C)−1 define a hard credal partition [21] of the training set T. In the following, we will derive the consequent class of belief rule $R^{j}$ by combining the class information of patterns in subset $T^{j},j=1,2,\cdots ,F\left(C\right)-1$.

First, for any pattern $x_{i}\in T^{j}$, we calculate the matching degree with antecedent part of belief rule $R^{j}$ using the geometric mean operator as

(11) $$\mu_{A^{j}}\left(x_{i}\right)=\sqrt[{P}]{\prod_{p=1}^{P}\mu_{A_{p}^{j}}\left(x_{ip}\right)},$$

where $\mu_{A_{p}^{j}}$ is the membership function of the fuzzy set $A_{p}^{j}$ defined in Equation (9).

Then, assume the class label of pattern $x_{i}$ is $c_{k}$, which takes value in class set C. This can be regarded as a piece of evidence that supports the consequent class belonging to $c_{k}$. However, this piece of evidence is not full certainty. In belief function theory, this can be expressed by saying that only some part of the belief (measured by the matching degree $\mu_{A^{j}}\left(x_{i}\right)$) is committed to $c_{k}$. Because $\mathrm{Class}\left(x_{i}\right)=c_{k}$ does not point to any other particular class, the rest of the belief should be assigned to the frame of discernment C representing global ignorance. Therefore, this item of evidence can be represented by a mass function $m^{j}\left(\cdot |x_{i}\right)$ verifying:

(12) $$\left\{\begin{array}{lll} m^{j}\left(\left\{c_{k}\right\}|x_{i}\right) & = & \mu_{A^{j}}\left(x_{i}\right)\\ m^{j}\left(C|x_{i}\right) & = & 1-\mu_{A^{j}}\left(x_{i}\right)\\ m^{j}\left(A|x_{i}\right) & = & 0,\;\;\forall A\in 2^{C}\backslash \left\{C,\left\{c_{k}\right\}\right\} \end{array}.\right.$$

Finally, the mass functions derived from all the patterns in $T^{j}$ are combined to obtain the consequent class of belief rule $R^{j}$. As the items of evidence from different labeled patterns are collected independently, the Dempster’s rule of combination is used in this work to synthesize the final consequent class membership as

(13) $$m^{j}=\underset{x_{i}\in T^{j}}{⨁}m^{j}\left(\cdot |x_{i}\right).$$

Noting that all the pieces of evidence have only one focal set except the global set C, the computation of Dempster’s rule is quite efficient. The belief degrees of the consequent class of rule $R^{j}$ are then obtained as $\beta_{k}^{j}=m^{j}\left(\left\{c_{k}\right\}\right),k=1,2,\cdots ,M$.

#### 3.2.3. Rule Weights Generation

As in [18], the rule weights can be derived based on two concepts called *confidence* and *support*, which are often used for evaluating association rules in data mining fields. The confidence is a measure of the validity of one rule, which is defined for belief rule as

(14) $$c\left(R^{j}\right)=1-\bar{K^{j}},$$

where $0\leq \bar{K^{j}}\leq 1$ is the average conflict factor, which measures the conflict among those pieces of evidence used for building the consequent class of rule $R^{j}$:

(15) Kj¯=0,if|Tj|=1,1|Tj|(|Tj|−1)∑xp,xq∈Tj;c(p)≠c(q)μAj(xp)μAj(xq),otherwise.

with $|T^{j}|$ donating the number of training patterns in *j*-th hard credal partition.

On the other hand, the support indicates the grade of the coverage by one rule, which is defined as the ratio of the number of covered patterns to the total pattern number:

(16) $$s\left(R^{j}\right)=\frac{|T^{j}|}{N}.$$

Based on the above two measures, the rule weights are finally derived as

(17) $$\theta^{j}=\frac{c\left(R^{j}\right)s\left(R^{j}\right)}{\max_{j}\left\{c\left(R^{j}\right)s\left(R^{j}\right),j=1,\cdots ,F\left(C\right)-1\right\}},\;\;j=1,2,\cdots ,F\left(C\right)-1.$$

### 3.3. Parameter Optimization for Trade-Off between Accuracy and Interpretability

In the above BRB learning process, the number of clusters *C* plays a key role in determining the accuracy and the interpretability of the learned classification model. Many clusters means many rules, which usually leads to high classification accuracy, but degrades the model’s interpretability. Therefore, we need to search for an optimal number of clusters to get the desired trade-off between accuracy and interpretability.

On the one hand, to obtain a model with high accuracy, the following leave-one-out test (mean squared) error *MSE* should be minimized:

(18) $$MSE=\frac{1}{N}\sum_{i=1}^{N}\sum_{j=1}^{M}{\left(P^{\left(i\right)}\left(\left\{\omega_{j}\right\}\right)-t_{j}^{\left(i\right)}\right)}^{2},$$

where $P^{\left(i\right)}\left(\left\{\omega_{j}\right\}\right)$, j=1,⋯,M, are the output of the belief reasoning method for training pattern $x^{\left(i\right)}$, and $t_{j}^{\left(i\right)}$, j=1,⋯,M, are binary indicator variables defined by $t_{j}^{\left(i\right)}=1$, if the real label of training pattern $x^{\left(i\right)}$ is $\omega_{j}$ and $t_{j}^{\left(i\right)}=0$, otherwise.

On the other hand, to obtain a model with high interpretability, the number of rules or, equivalently, the number of clusters should be minimized. However, the number of clusters should not be too small to ensure the cluster validity. To assess the quality of fuzzy partitions, a great number of validity indexes have been proposed in the literature [26,27,28,29]. One of the representatives is the fuzzy partition entropy *FPE* [30] defined by

(19) $$FPE=\frac{1}{N\log_{2}\left(C\right)}\sum_{i=1}^{N}\sum_{j=1}^{C}\mu_{ij}\log_{2}\left(\frac{1}{\mu_{ij}}\right),$$

where $\mu_{ij}$ is the membership degree of *i*-th pattern in *j*-th cluster. The optimal number of clusters *C* is obtained by minimizing *FPE* with respect to $C=2,3,\cdots ,C_{\max}$.

The above fuzzy entropy-based validity index has inspired us to use similar definitions of entropy in belief function framework to assessing the quality of evidential partitions. The definition of entropy in belief function framework has been a hot research subject in the past few years [31,32,33]. A representative one is the aggregated entropy *AE* introduced by Pal et al. [34], which satisfies natural requirements and has interesting properties. It is defined for a *normal* mass function *m* as

(20) $$AE\left(m\right)=\sum_{A\in F(m)}m\left(A\right)\log_{2}\left(\frac{\left|A\right|}{m(A)}\right),$$

where F(m) denotes the set of focal sets of *m*. This entropy measure can be further decomposed as the sum of two terms:

(21) $$AE\left(m\right)=\sum_{A\in F(m)}m\left(A\right)\log_{2}\left|A\right|+\sum_{A\in F(m)}m\left(A\right)\log_{2}\left(\frac{1}{m(A)}\right).$$

The first term is the nonspecificity measure, which reflects the degree of imprecision of *m*, whereas the second term reflects the inconsistency in *m* and can be seen as a measure of conflict. Therefore, AE(m) tends to be small when the mass is assigned to few focal sets, with small cardinality.

Though the above *AE* measure was defined for *normal* mass functions (i.e., *m* with m(∅)=0), it can be easily extended to *subnormal* mass functions considered in the paper by defining the cardinality of the empty set as C (This extension is justified by the fact that the mass given to the empty set corresponds to a situation of maximal uncertainty, just like the mass given to Ω [35].). The evidential partition entropy *EPE* is then defined as the average *AE* as

(22) $$EPE=\frac{1}{N\log_{2}\left(C\right)}\sum_{i=1}^{N}\sum_{A\in F(m_{i})}m_{i}\left(A\right)\log_{2}\left(\frac{\left|A\right|}{m_{i}\left(A\right)}\right).$$

When all the patterns are assigned to singleton sets $\emptyset ,\omega_{1},\omega_{2},\cdots ,\omega_{C}$, EPE gets the lower bound value 0. The maximum value of EPE is attained for $m_{i}$ such that $m_{i}\left(A\right)\propto \left|A\right|$, for all $A\in F(m_{i})$. It should also be noted that the fuzzy partition entropy *FPE* defined in Equation (19) is a special case of our defined evidential partition entropy *EPE* when each $m_{i}$ is a *Bayesian* mass function.

Finally, a single scalar objective function for the number of clusters *C* is then defined based on the above two objectives *MSE* and *EPE* as

(23) J(C)=λ·MSE+(1−λ)·EPE,

where λ∈[0,1] is the weight characterizing the user’s preference for classification accuracy. When λ=1, the classification accuracy is the only objective, whereas when λ=0, only the cluster validity is guaranteed. With given weight λ, by minimizing the above objective function, an optimal number of clusters *C* can be obtained for a better trade-off between accuracy and interpretability.

## 4. Experiments

The performance of the proposed CBRBCS was assessed by two different types of experiments. In the first experiment, a synthetic data set was used to show the behavior of the proposal in controlled settings. In the second one, 20 real data sets from the UCI Machine Learning Repository [23] were considered, with the aim to show that the proposed technique is adequate for a variety of real tasks.

### 4.1. Synthetic Data Set Test

A two-dimensional four-class synthetic data set was designed to illustrate the interest of the compact BRB learning method in CBRBCS. The following normal class-conditional distributions were assumed:

Class $\omega_{1}$: $\mu_{1}={\left(0,0\right)}^{T},\;\Sigma_{1}=2I$;  Class $\omega_{2}$: $\mu_{2}={\left(5,0\right)}^{T},\;\Sigma_{2}=2I$;

Class $\omega_{3}$: $\mu_{3}={\left(2,5\right)}^{T},\;\Sigma_{3}=2I$;  Class $\omega_{4}$: $\mu_{4}={\left(3,5\right)}^{T},\;\Sigma_{4}=2I$.

**Figure 2:**
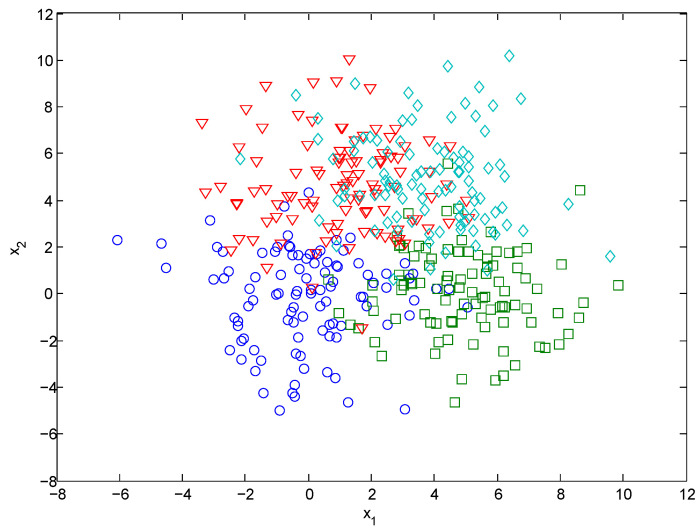
Synthetic data set (‘◯’ for class $\omega_{1}$, ‘□’ for class $\omega_{2}$, ‘▽’ for class $\omega_{3}$ and ‘◊’ for class $\omega_{4}$).

A set of 400 samples was generated from the above distributions using equal prior probabilities. This data set is displayed in Figure 2. The proposed method was used to learn BRBs from this data set. The default values of open parameters in ECM were used and different values of the accuracy weight λ were considered for comparison.

**Figure 3:**
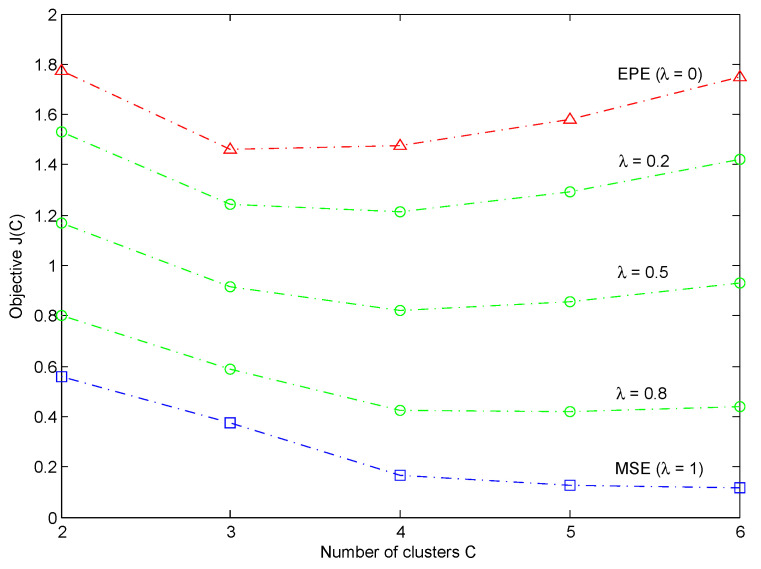
Objective values J(C) of the learned BRBs under different numbers of clusters *C*.

Figure 3 shows the objective values J(C) of the learned BRBs under different numbers of clusters (C=2,3,4,5,6). When the accuracy weight λ=1, the objective function J(C) just reduces to the *MSE* measure. It can be seen that as the increasement of the number of clusters (or, equivalently, the number of rules), the *MSE* decreases gradually. By minimizing the *MSE*, a large BRB is obtained with high classification accuracy. By contrast, when the accuracy weight λ=0, the *EPE* measure is recovered. We see that the *EPE* reaches its minimal value when the number of clusters equals to 3, and after that it increases as the increasement of the number of clusters. In the same way of minimizing the *EPE*, we can get a small BRB with higher model interpretability, but relatively lower classification accuracy. Finally, when the accuracy weight 0<λ<1, the objective value J(C) provides a trade-off between the *MSE* and the *EPE*. Please note that the three considered weights (λ=0.2,0.5, and 0.8) give the same decision for the optimal number of clusters as C=4, in which case, a total number of $(C^{2}+C)/2+1=11$ belief rules are learned with classification accuracy of 83.55%.

### 4.2. Real Data Set Test

In this experiment, 20 representative real data sets from UCI Machine Learning Repository were selected to evaluate the performance of the proposed CBRBCS. The main characteristics of the 20 data sets are summarized in Table 2. It can be seen that the selected data sets vary greatly in the number of instances (from 80 to 12,690), the number of features (from 4 to 60), and the number of classes (from 2 to 11).

To develop the experiment, we consider the *B-Fold Cross-Validation* (B-CV) model. Each data set is divided into *B* blocks, with B−1 blocks as a training set and the remaining block as a test set. Therefore, each block is used exactly once as a test set. We use the 10-CV model here, i.e., ten random partitions of the original data set, with nine of them (90%) as the training set and the remainder (10%) as the test set. For each data set, we consider the average results of the ten partitions.

The performance of the proposed classifier is compared with the traditional BRBCS [18] as well as several other representative classifiers, including K-NN (instance-based classifier) [2], C4.5 (decision tree-based classifier) [3], SVM (statistical classifier) [4], and FRBCS (rule-based classifier) [5]. Settings of these comparison methods are summarized in Table 3.

**Figure 4:**
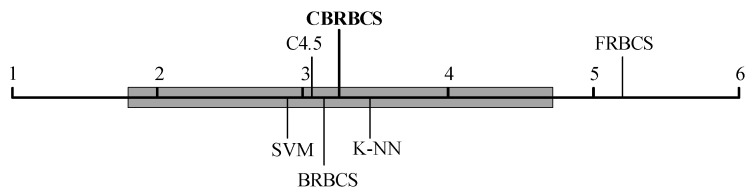
Bonferroni-Dunn test result of the average accuracy ranks.

Table 4 shows the classification accuracy rates of different methods for real data sets. The numbers in brackets represent the ranks of the classification accuracy for each method and the last row shows the average ranks of all the methods over the 20 data sets. It can be seen that the performance of the two belief rule-base classifiers, i.e., BRBCS and CBRBCS, is comparable with those classical methods. To compare the classification results statistically, we carry out nonparametric tests [36,37] for multiple comparisons based on the average accuracy ranks obtained over the considered data sets. First, we use the Iman-Davenport test to determine whether significant differences exist among different methods. The Iman-Davenport statistic (distributed according to the F-distribution with k−1=5 and (k−1)(N−1)=95 degrees of freedom, where *k* is the number of compared methods and *N* is the number of data sets) is 4.99 for average ranks and the corresponding critical value is 2.29 for a significance level of α=0.05. Given that the Iman-Davenport statistic is clearly greater than the critical value, the test rejects the null hypothesis, and therefore, it can be said that there are significant differences among the accuracy results of the considered methods. Then, we apply the *post hoc* Bonferroni-Dunn test to compare the control method (i.e., the proposed CBRBCS) with the remaining ones. Figure 4 shows the test result of the average accuracy ranks with a significance level of α=0.05, in which case the calculated critical difference is 1.52. The critical difference value is represented as a thicker horizontal line, and those values that exceed this line are methods with significantly different results from the control method. It can be seen that the proposed CBRBCS performs significantly better than the FRBCS, and obtains similar classification accuracy with the traditional BRBCS. Compared with other non-rule-based classifiers including SVM, C4.5 and K-NN, although the classification accuracy differences among them are not very significant, the proposed CBRBCS is preferable as it can provide a more interpretable classification model on the premise of comparative accuracy.

To evaluate the interpretability of the classification models, Table 5 displays the numbers of generated rules for the two belief rule-based methods, i.e., BRBCS and CBRBCS. It can be seen that for all the evaluated data sets, much smaller number of rules are generated for the proposed CBRBCS. To show the rule reduction performance more clearly, we also provide the *rule reduction rate* (defined as $\left(\#{\mathrm{Rule}}_{\mathrm{BRBCS}}-\#{\mathrm{Rule}}_{\mathrm{CBRBCS}}\right)/\#{\mathrm{Rule}}_{\mathrm{BRBCS}}$) in the last column. We can notice that for those data sets with large numbers of train instances and features, but small number of classes (like Australian, Car, Contraceptive, Ionosphere, Nursery, Sonar, Thyroid, Vehicle), the proposed CBRBCS achieves more significant rule reduction performance (with rule reduction rate >90%). The reason is that in the traditional BRBCS, the rules are generated based on fuzzy-grid method, in which case, the number of generated rules is positive correlated with both the numbers of train instances and features. However, the number of generated rules for the clustering-based learning method used in CBRBCS is only determined by the underling structure of the data, which is closely related to the number of classes. Therefore, compared with the traditional BRBCS, the proposed CBRBCS obtains a better trade-off between accuracy and interpretability (similar classification accuracy is obtained with much smaller number of rules).

## 5. Conclusions

In this paper, a compact belief rule-based classification system with ECM clustering has been proposed to overcome the limitations of the traditional BRBCS in large data set conditions. Instead of defining belief rules for individuals of the training patterns, belief rules are constructed based on credal partitions of the training set. The two-objective optimization procedure based on both the mean squared error and the evidential partition entropy can successfully find an optimal number of clusters. This method can discover the underlying data structure, which can be successfully translated into belief rules. From the results reported in the last section, we can conclude that the proposed technique can obtain a better trade-off between accuracy and interpretability than the traditional one. Furthermore, compared with other non-rule-based classifiers, the proposed technique can obtain competitive classification performance in accuracy. Therefore, this technique is be a better choice for those classification problems where both high accuracy and interpretability are needed.

## Figures and Tables

**Table 1:** List of symbols and definitions.

Symbol	Definitions
AE	aggregated entropy
BRB	belief rule base
BRBCS	belief rule-based classification system
BRM	belief reasoning method
CBRBCS	compact belief rule-based classification system
ECM	evidential C-means
EPE	evidential partition entropy
FCM	fuzzy C-means
FPE	fuzzy partition entropy
FRBCS	fuzzy rule-based classification system
K-NN	K-nearest neighbor
MSE	mean squared error
RBC	rule-based classification
SVM	support vector machines
$A^{j}$	antecedent part of belief rule $R^{j}$
C	set of classes
*c*	class label
*C*	number of clusters
$d_{ij}$	distance between object $x_{i}$ and set $A_{j}$
*M*	credal partition matrix
$n_{p}$	number of partitions for *p*-th feature
$R^{j}$	*j*-th belief rule in the rule base
T	training data set
*V*	cluster center matrix
*W*	weight of class labels in clustering process
x	input feature vector
α	weighting exponent for cardinality in ECM
β	weighting exponent for fuzziness in ECM
δ	distance to the empty set in ECM
$\theta^{j}$	weight of belief rule $R^{j}$
λ	weight of classification accuracy
$\sigma_{p}^{2}$	variance of *p*-th feature values
$\sigma_{c}^{2}$	variance of class values
Ω	frame of discernment

**Table 2:** Statistics of the real data sets used in the experiment.

Data Set	# Instances	# Features	# Classes
Australian	690	14	2
Balance	625	4	3
Car	1278	6	4
Contraceptive	1473	9	3
Dermatology	358	34	6
Ecoli	336	7	8
Glass	214	9	6
Hepatitis	80	19	2
Ionosphere	351	33	2
Iris	150	4	3
Lymphography	148	18	4
Nursery	12,690	8	5
Page-blocks	5472	10	5
Sonar	208	60	2
Thyroid	7200	21	3
Vehicle	846	18	4
Vowel	990	13	11
Wine	178	13	3
Yeast	1484	8	10
Zoo	101	16	7

**Table 3:** Settings of the comparison methods.

Method	Parameter	Value
K-NN	number of neighbors *K*	3
	distance metric	Euclidean
C4.5	pruned?	TRUE
	confidence level *c*	0.25
	minimal instances per leaf *i*	2
SVM	kernel type	RBF
	penalty coefficient *C*	100
	kernel parameter γ	0.01
FRBCS	number of partitions per feature *n*	5
	membership function type	triangular
	reasoning method	single winner
BRBCS	number of partitions per feature *n*	5
	membership function type	triangular
	reasoning method	belief reasoning
CBRBCS	weighting exponent for cardinality α	2
	weighting exponent for fuzziness β	2
	accuracy weight λ	0.5

**Table 4:** Classification accuracy rates (in %) for real data sets.

Data set	K-NN	C4.5	SVM	FRBCS	BRBCS	CBRBCS
Australian	88.78 (1)	85.22 (2)	75.51 (6)	79.86 (5)	82.74 (4)	83.90 (3)
Balance	83.37 (5)	76.80 (6)	95.51 (1)	89.60 (4)	92.66 (3)	93.20 (2)
Car	92.31 (6)	92.55 (5)	94.33 (2)	92.78 (4)	95.23 (1)	93.12 (3)
Contraceptive	44.95 (5)	52.68 (2)	55.95 (1)	39.86 (6)	49.15 (3)	48.20 (4)
Dermatology	96.90 (1)	94.42 (2)	94.34 (3)	72.29 (6)	85.12 (5)	93.35 (4)
Ecoli	80.67 (3)	79.47 (4)	81.96 (2)	76.02 (6)	78.34 (5)	82.62 (1)
Glass	70.11 (1)	67.44 (5)	70.00 (2)	66.04 (6)	69.04 (3)	68.15 (4)
Hepatitis	82.51 (3)	84.00 (1)	82.18 (4)	74.41 (6)	76.28 (5)	83.68 (2)
Ionosphere	85.18 (6)	90.90 (3)	92.60 (1)	86.55 (5)	89.11 (4)	91.66 (2)
Iris	94.00 (4)	96.00 (3)	97.33 (1)	93.67 (5)	96.67 (2)	93.33 (6)
Lymphography	77.39 (4)	74.30 (5)	81.27 (1)	72.27 (6)	79.20 (2)	77.90 (3)
Nursery	92.54 (6)	97.30 (1)	93.18 (5)	94.02 (4)	96.05 (2)	94.65 (3)
Page-blocks	95.91 (2)	96.97 (1)	92.36 (5)	91.92 (6)	95.10 (4)	95.28 (3)
Sonar	83.07 (1)	70.07 (5)	78.71 (2)	59.60 (6)	74.80 (3)	73.33 (4)
Thyroid	93.89 (5)	99.63 (1)	93.49 (6)	94.03 (4)	95.04 (2)	94.39 (3)
Vehicle	71.75 (3)	74.69 (1)	52.95 (6)	60.77 (5)	71.95 (2)	70.54 (4)
Vowel	97.78 (1)	81.52 (5)	95.76 (2)	79.90 (6)	93.28 (3)	92.10 (4)
Wine	95.49 (3)	94.90 (4)	89.74 (6)	95.82 (2)	96.14 (1)	94.48 (5)
Yeast	53.17 (5)	55.53 (2)	58.09 (1)	48.51 (6)	54.66 (4)	55.08 (3)
Zoo	92.81 (4)	93.64 (3)	96.50 (1)	85.06 (6)	90.55 (5)	95.30 (2)
Average rank	3.45	3.05	2.90	5.20	3.15	3.25

**Table 5:** Numbers of generated rules of BRBCS and CBRBCS for real data sets.

Data Set	# Train Instances	# Features	# Classes	# Rules		
				BRBCS	CBRBCS	Reduction Rate
Australian	621	14	2	317	16	94.95%
Balance	552	4	3	66	11	83.33%
Car	1150	6	4	682	22	96.77%
Contraceptive	1326	9	3	233	22	90.56%
Dermatology	322	34	6	315	37	88.25%
Ecoli	302	7	8	45	37	17.78%
Glass	192	9	6	40	22	45.00%
Hepatitis	72	19	2	67	7	89.55%
Ionosphere	316	33	2	227	11	95.15%
Iris	135	4	3	14	11	21.43%
Lymphography	133	18	4	129	22	82.95%
Nursery	11,421	8	5	4238	37	99.13%
Page-blocks	4925	10	5	55	22	60.00%
Sonar	187	60	2	187	16	91.44%
Thyroid	6480	21	3	460	22	95.22%
Vehicle	761	18	4	230	16	93.04%
Vowel	891	13	11	141	67	52.48%
Wine	160	13	3	122	16	86.89%
Yeast	1336	8	10	96	56	41.67%
Zoo	91	16	7	55	29	47.27%
